# Categorization of screen glasses of mobile devices with respect to their EPR spectral properties and potential applicability for use in retrospective dosimetry

**DOI:** 10.3389/fpubh.2025.1659601

**Published:** 2025-09-09

**Authors:** Agnieszka Marciniak, Małgorzata Juniewicz, Bartłomiej Ciesielski, Piotr Boguś, Kamila Mazur-Oleszczuk, Anita Prawdzik-Dampc, Jakub Karczewski

**Affiliations:** ^1^Department of Physics and Biophysics, Medical University of Gdańsk, Gdańsk, Poland; ^2^Division of Artificial Intelligence, Institute of Informatics, Faculty of Mathematics, Physics and Informatics, University of Gdańsk, Gdańsk, Poland; ^3^Department of Oncology and Radiotherapy, Medical University of Gdańsk, Gdańsk, Poland; ^4^Institute of Nanotechnology and Materials Engineering, Faculty of Applied Physics and Mathematics, Gdańsk University of Technology, Gdańsk, Poland

**Keywords:** EPR, glass, retrospective dosimetry, radiation accident, mobile devices

## Abstract

**Introduction:**

Industrial and medical applications of ionizing radiation, as well as unstable political situation worldwide, which may result in military releases of radioactivity, increase a risk of uncontrolled exposures of people to ionizing radiation. Retrospective dosimetry allowing for fast triage of victims is crucial for rescue actions. Previous studies showed that smartphones’ screens are promising for dosimetry based on electron paramagnetic resonance (EPR). A variety of EPR line-shapes in different screens, regarding background signals (BG) and radiation-induced signals (RIS), various sensitivities to interfering factors like UV light and temperature, impose serious limitations on this method. This study focuses on classification of screen glasses, taking into account their elemental compositions, EPR properties (sensitivity to UV and temperature), in order to formulate practical recommendations for dosimetry.

**Methods:**

EPR spectra of 45 screens, unirradiated and irradiated with X-rays, were measured. Elemental composition of the glasses was determined using Energy Dispersive Spectroscopy. Effects of UV on samples’ EPR spectra were checked. Annealing at 200 °C enabled to evaluate effects of heating on BG and RIS. A self-written program based on c-means algorithm was used to find intercorrelations between elemental compositions and EPR features (types) of the glasses.

**Results and discussion:**

Our spectra-differentiating algorithm resulted in identification of five types of glasses correlated with their elemental composition, sensitivity to X-rays, to UV and high temperature. Glasses labelled as type III and V were recommended for dosimetry due to their resistance to UV and undergoing temperature-caused bleaching of RIS without affecting their BG signals; a feature which enables reconstruction of the original BG from an irradiated sample – a key step in retrospective dosimetry. The introduced categorization of screen glasses, based on chosen features of their EPR spectra, is a simple and practical method for evaluation of their applicability in retrospective dosimetry following radiation accidents, e.g. for triage of exposed people.

## 1 Introduction

The awareness of society that people can be exposed to hazardous effects of ionizing radiation is growing. The current political situation in the world leading to a growing threat of using nuclear weapons as well as the continuous development of industrial and medical applications of ionizing radiation, increases a risk of potential exposure of people to uncontrolled doses of ionizing radiation. Therefore, the research on reliable methods of accidental dosimetry is ongoing, including a search for new materials potentially applicable for retrospective personal EPR dosimetry. Such material should be present in the closest human environment, should show high sensitivity to ionizing radiation and stability of the dosimetric signal, its collection should be noninvasive and the measurement should allow for a reliable dose reconstruction in the shortest possible time, which would allow for triage of the exposed people and undertaking the necessary medical actions. To date a number of retrospective dosimetry studies on solid biological materials, such as teeth (1–3), bones (4), hair (5), nails (6) as well as some artificial materials from the human environment, such as plastics (7), cotton wool, ceramics, sacharides (8), glass (3, 9–12), eyeglasses (13), sorbitol (14) have been published. Aboelezz et al. (15) presented dosimetric characteristics of newly developed nanomaterials and hybrid composites applicable in EPR dosimetry. Updated and comprehensive summary of materials applicable for retrospective dosimetry based not only on EPR, but also on other methods (TL, OSL) was published by Yang et al. (16). Recently the research has focused on glass from mobile phones, which are ubiquitous in human environment (17, 18).

So far several aspects of EPR dosimetry in glasses from mobile devices have been studied, mainly related to decay of the dosimetric signal over time after irradiation (19), changes in the EPR signal related to sample preparation – crushing and water treatment (10, 11, 19) and influence of external physical factors such as light (20, 21) and temperature (22–25). Although initial results are promising, more detailed studies need to be conducted on the effect of UV light on EPR signal, in particular those in which UV light generates an additional EPR spectral component – light induced signal (LIS) and therefore affects reliable determination of the radiation induced signal (RIS) component. Our previous studies regarding the effects of heating on generation of heat-induced signals (HIS) and on elimination of the RIS (25) confirmed usefulness the heating method for reconstruction of the native BG signal in irradiated samples, which is crucial in EPR dosimetry.

The main problem in accidental EPR dosimetry with glasses is caused by variety of line shapes of their native background signals (BG) in various glasses. In order to reconstruct the absorbed dose from measured EPR spectra, information about the background signal is necessary. This crucial requirement is difficult to fulfill in a real scenario, when the sample irradiated during an accident is the only one available (18, 19). Applicability of annealing of irradiated samples for reconstruction of their BG signal was proved for watch glasses (25), however, this method has not been verified for mobile phone screen glass. Effects of sample heating on its EPR signal were also presented by other researchers (10, 20, 23, 26, 27).

Until now some researchers have also attempted to categorize glasses into individual types on the basis their of EPR lines. Trompier et al. (28) distinguished 5 types of spectral line shape, but the tested phones came from early years of production (up to 2012) and only 24 out of 75 had a touch screen. However, Bassinet et al. (10) examined 20 displays of mobile phones, among which they distinguished two types of spectral line-shape.

In this article, the classification of glass EPR spectra from 45 screens was based on their spectral features at chosen g-values and resulted in grouping them into five types of EPR line shapes. It was shown, that the assignment of a spectrum to one of the 5 categories can be performed automatically using a custom-developed sorting algorithm. Moreover, the elemental composition of the glasses was also examined and radiation sensitivity of the samples (i.e., the quantitative response of spectra amplitudes to the dose) was determined. Correlations between those characteristics were analysed using clustering c-means algorithm (29, 30).

The aim of the glass categorization was to indicate the most applicable glasses for reliable EPR dosimetry based on the correlation of the shape of the EPR spectral line with other glass features, including the presence or absence of parasitic signals generated by light and heating at high temperatures (≥ 200 °C).

## 2 Materials and methods

### 2.1 Samples

The samples were obtained from touch screens of 45 electronic devices – 44 mobile phones (39 different models) and one tablet (Table 1). Two phones were purchased as new for this project and the rest devices were used for various periods of time and were donated for this study by their owners. One phone was produced in 2009, 3 phones in 2012 (type I), 9 phones and the tablet were produced in 2013–2014 (type I and III) and 26 phones produced in 2015–2019. For 5 phones we have no information regarding the date of production. All those mobile phones were touch screen devices. Screen glass of 19 out of 45 devices was Corning Gorilla Glass, according to technical specifications (www.gsmarena.com, www.mgsm.pl).

**Table 1:** Elemental composition of the tested glasses (excluding the content of oxygen).

Name of sample	Content of elements in %						
	Si	K	Mg	Al	Na	Ca	P
I_1 norm (*)	**50**	**19.3**	**3.4**	**19.3**	**8.0**		
I_2 norm	**54.0**	**20.7**	**4.6**	**14.9**	**4.6**	**1.1**	
I_3	52.8	24.2	2	16.2	4.8		
I_4	56	21.8	2.5	15	4.7		
I_5	55.6	25.1	0.6	17.9	0.8		
I_6 (*)	53	23.7	2	16.7	4.6		
I_7	55.6	22.4	2.4	16.2	3.4		
I_8	56.5	22.4	0.6	17.8	2.7		
I_9	56.2	21.1	2.5	15.1	5.1		
I_10	53.6	20.2	0.7	23.2	2.3		
I_11 (*)	53.2	23.9	2.1	16.3	4.5		
I_12	55.6	23.8	0.6	18.1	1.9		
I_13	55.9	20.2	2.5	15.6	5.8		
I_14	55.9	21.8	2.5	15.2	4.6		
I_15	53.4	23	2	16.2	5.4		
I_16	56.5	24	3	12.5	3	1	
II_1 norm	**54.2**	**16.9**	**1.2**	**21.7**	**6.0**		
II_2	**55.8**	**24**	**0.5**	**18**	**1.7**		
II_3	55.9	23.9	0.6	18.2	1.4		
II_4	55.5	24	0.6	18.2	1.7		
III_1 norm	**59.6**		**5.6**	**2.2**	**24.7**	**7.9**	
III_2 norm (×)	**58.4**	**14.6**	**4.5**	**5.6**	**9.0**	**7.9**	
III_3	62.2	14.7	3.7	2	5.9	11.5	
III_4	64.2	8	4.1	1.9	9.8	12	
III_5	64.8	4.1	3.5	5.6	11.7	10.3	
III_6	57.7	20.6	3.5	4.6	3.4	10.2	
III_7	61.2	16.5	4.2	1.9	5	11.2	
III_8 (×)	58.3	16.4	3.4	4.3	7.1	10.5	
III_9	60.6	19.4	3.3	2.5	2.1	12.1	
III_10	62.8	9.7	4.1	2.3	9.7	11.4	
III_11	58.5	17.8	3.3	4.3	5.3	10.8	
III_12	63.1	0.6	3.8	4.4	17.1	11	
III_13	62.7	11.6	3.6	2.2	7.1	12.8	
IVA_1 norm (#)	**38.6**	**15.9**	**2.3**	**22.7**	**10.2**		**10.2**
IVA_2	40.3	21.2	0.5	19.8	4.6		13.6
IVB_1 (#)	40.2	19.8	0.7	20.4	5.2		13.7
IVB_2	40.4	17.4	0.7	19.9	7.9		13.8
V_1 norm	**49.5**	**13.2**	**7.7**	**15.4**	**14.3**		
V_2 (+)	52.9	24.9	5.5	12.3	4.4		
V_3 (+)	53.6	26.7	5.2	12.2	2.3		
V_4	51.8	25.5	5.4	13.6	3.7		
V_5	57.5	19.8	3.7	14.7	4.3		
V_6	54.4	18.3	5.5	13.4	8.4		
V_7	53	25	5.2	12.8	3.1		
V_8	53	24.9	5.8	11.8	4.5		

The same phone models were marked as: (*) – Samsung Galaxy Ace 4 (line-shape I), (×) – Microsoft Mobile RM-1127 (Microsoft Lumia 550) (line-shape III), (#) – Microsoft Lumia 950XL (line-shape IV) and (+) – Huawei P8 Lite (ALE-L21) (line-shape V). The bold font refers to the data normalised to 100% for the total of 7 elements (i.e. all but oxygen).

Elemental composition of the glasses was determined by Energy Dispersive Spectroscopy (EDS) at the institute of Nanotechnology and Materials Engineering of the Gdańsk University of Technology. The measurement uncertainty was 0.5% for all measured elements’ concentrations.

### 2.2 EPR measurements

EPR measurements were performed at room temperature using a Bruker EMX-6/1 (Bruker BioSpin) spectrometer in X-band (9.85 GHz) with cylindrical cavity 4119HS W1/0430. The 110–160 mg glass samples were measured in a quartz EPR sample tube of 4 mm inner diameter. EPR spectra were recorded with the following acquisition parameters: 350.5 mT central magnetic field, 100 kHz modulation frequency, 0.15 mT modulation amplitude, 32 mW microwave power, 163.84 ms time constant, 335.54 s sweep time, 5 averaged scans per one spectrum. Each sample was measured at two orientations of the sample tube in the cavity and the spectra were averaged and normalized to the sample mass. Intracavity manganese standard sample (Mn^2+^ in MgO) was measured simultaneously with all samples and used to calibrate the magnetic field – the two sharp manganese lines can be seen at the spectra wings. All measured spectra were aligned along magnetic field axis with respect to the standard lines, normalized to the amplitude of the standard lines. The spectrum of empty EPR tube was subtracted and linear baseline correction was applied to all spectra.

### 2.3 Irradiations

The samples were irradiated at room temperature under electron equilibrium conditions at the Department of Oncology and Radiotherapy, Medical University of Gdańsk, Poland, using X-rays from TrueBeam SN 2400 medical accelerator. The samples were placed on 30x30x10 cm PMMA phantom under 1.5 cm of water-equivalent gel bolus material to assure the sample positioning at the maximum of the depth-dose distribution for X-rays generated at 6 MV voltage. The dose delivered to all samples was 10 Gy (in terms of dose to water) under electronic equilibrium conditions, with uncertainty of 2%.

### 2.4 Illumination

The artificial UV light was generated by a lamp made of two parallel CLEO advantage 80 W-R bulbs (Philips) with a power of 80 W each - the spectrum was presented in Juniewicz et al. (21).

### 2.5 Annealing of the samples

Annealing of unirradiated and irradiated samples was performed in a drying oven VWR VENTI-Line at 200 °C.

### 2.6 Data analysis

Quantitative analysis of the spectra (alignment and normalization of their amplitudes to the standard’s lines, subtractions of the empty tube spectrum, averaging) was carried out using Microsoft Office Excel 2010. Decomposition of the spectra into BG, RIS, LIS and HIS components was performed using REGLINP procedure in Excel. All calculations and graphical presentation of clustering c-means algorithm were performed using the program prepared in Matlab software.

## 3 Results

Table 1 presents elemental composition of analysed glasses. For some of the samples, marked as I_1_norm, I_2_norm, II_1_norm, III_1_norm, III_2_norm, IV_1_norm and V_1_norm, the percentage content of all 8 elements (Si, K, Mg, Al, Na, Ca, P and O) was measured, while for the other samples only 7 elements (Si, K, Mg, Al, Na, Ca, P) were measured. Therefore, for consistency of the presented data allowing for they comparison, the elemental compositions presented in Table 1 were normalized to 100% for the total of 7 elements (i.e., all but oxygen). The presence of calcium above 7% was observed only in samples representing shape III and the presence of phosphorus only in type IV samples.

In the examined 19 glass screens of Gorilla Glasses EPR spectra shapes of all types I-V were observed. Table 2 presents names of the screen models used in the experiments and their assignment to the types I–V. About 36% samples of type I, 29% samples of type III and 18% samples of type V were distinguished. Only four glasses were assigned to type II and two each to type IVA and IVB.

**Table 2:** Summary of all phone models used in this study, categorized by EPR spectral line-shapes.

Samples number	Shape I	Shape II	Shape III	Shape IVA	Shape IVB	Shape V
1	Samsung Galaxy Ace 4 (SM-G357FZ) (I)	**Samsung Galaxy XCover 4 (SM-G390F)**	**Samsung Galaxy GT-i9301i** **(Galaxy S3 Neo)**	**Microsoft Lumia 950XL (I)** **(RM-1116)**	**Microsoft Lumia 950XL (II)**	iPhone 6S
2	Samsung Galaxy mini 2	Samsung Galaxy S5 Neo G903F (SM-G903F)	Microsoft Mobile RM-1127 (Microsoft Lumia 550) (I)	**Sony Xperia XA1**	**Samsung Galaxy S6 Edge** **(SM-G925F)**	**Huawei P8 Lite (ALE-L21) (I)**
3	Motorola Moto G5	**HTC Desire 825**	myPhone Cube			**Huawei P8 Lite (ALE-L21) (II)**
4	LG	Xiaomi Redmi 6A (M1804C3CG)	**ASUS ZenFone 2 Laser 5.0 (ZE500KL)**			Motorola Moto E6 Plus
5	Xiaomi Redmi 6 (M1804C3DG)		**Samsung Galaxy J7 (SM-J730F/DS)**			Sony Xperia (GB/T 18287–2013)
6	Samsung Galaxy Ace 4 (SM-G357FZ) (II)		Huawei Ascend G620S			iPhone SE (A1723)
7	Samsung Galaxy Core Plus (SM-G350)		HTC beats audio			**Samsung Galaxy J3 DUOS (SM-J330F/DS)**
8	HUAWEI Y5 (DRA-L21)		Microsoft Mobile RM-1127 (Microsoft Lumia 550) (II)			ZTE Blade V7 LITE
9	**NOKIA Lumia 625 (RM-941)**		Lenovo Vibe K5 (A6020)			
10	Samsung Galaxy PO BOX 12987		**iPhone 5S (A1457)**			
11	Samsung Galaxy Ace 4 (SM-G357FZ) (III)		Asus ZenFone Go ZC500TG (X009DD)			
12	**HUAWEI P9 EVA-L09**		**Xiaomi Redmi Note 7 (M1901F7G)**			
13	**Sony Xperia J (ST26i)** **(PM-0160-BV)**		SMART			
14	**LG Optimus L9 (P760, Swift L9)**					
15	Tablet Samsung Galaxy Note 10.12014 LTE (SM-P605)					
16	**Nokia X6-00 (RM-559)**					

Gorilla Glass is marked by bold font.

**Figure 1:**
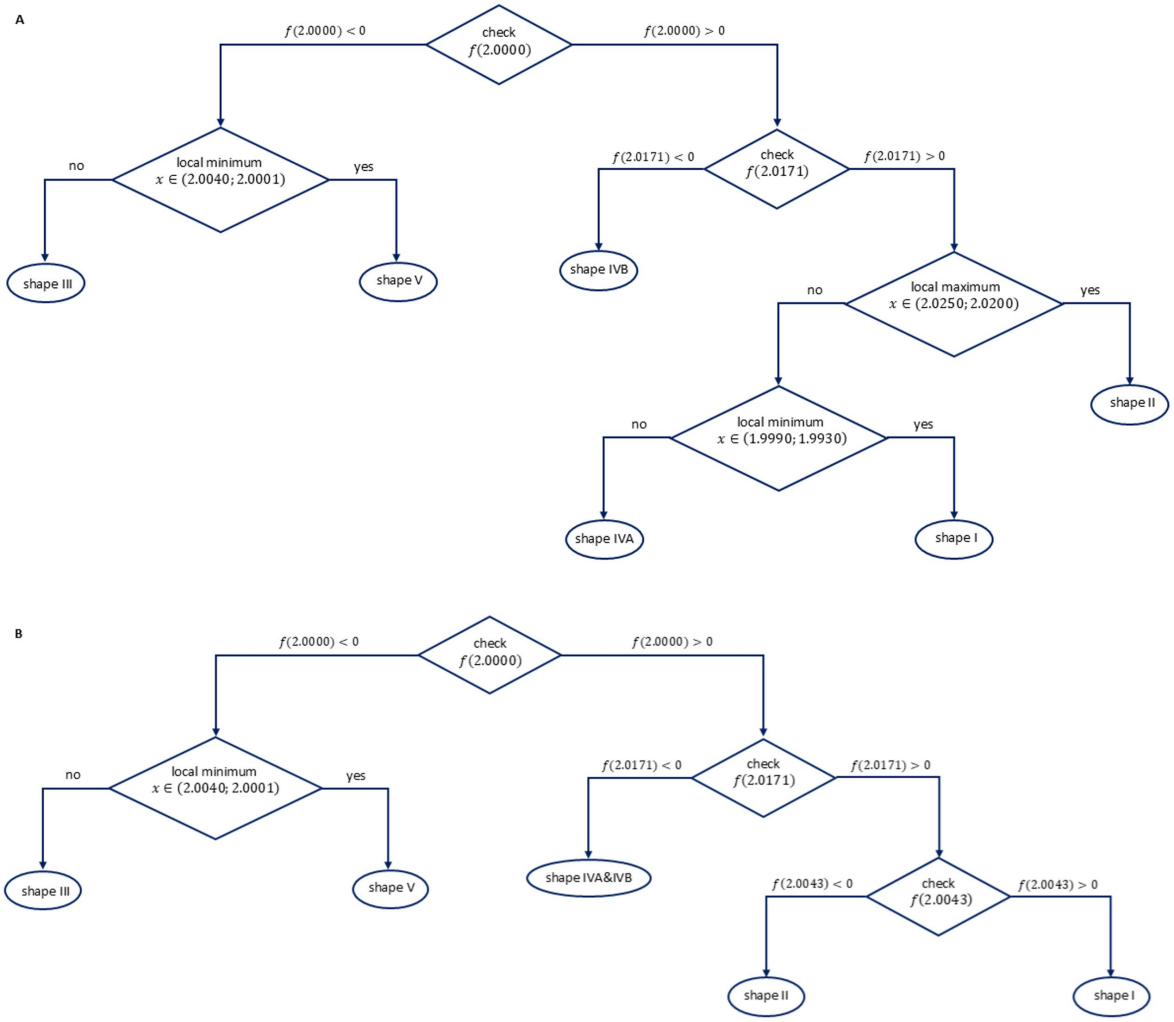
The sorting algorithm for **(A)** non-irradiated and **(B)** irradiated (10 Gy) glass spectra.

The spectra were analyzed and grouped into types I to V using the following properties: the range of the g-factors for positive and negative values of the spectral line and presence of local maximum and minimum within selected ranges of g values. Taking into account the above mentioned features, two sorting algorithms were prepared and the respective decision trees are presented in Figures 1A,B.

The root node and the decision nodes at the first depth are the same in both algorithms. At the second depth, the algorithm for non-irradiated samples distinguishes between shapes III, V and IVB. Similarly, the algorithm for irradiated samples distinguishes shape III and V from shapes IVA and IVB. For non-irradiated samples, two additional decision nodes are needed for the final spectral recognition: the decision node at the second depth (which distinguishes shape II) and the decision node at the third depth, which distinguishes shape I and shape IVA.

The algorithms were expressed using Excel functions to enable automatic categorization; they were tested for all 45 samples and correctly assigned the samples to the five line-shape types.

**Figure 2:**
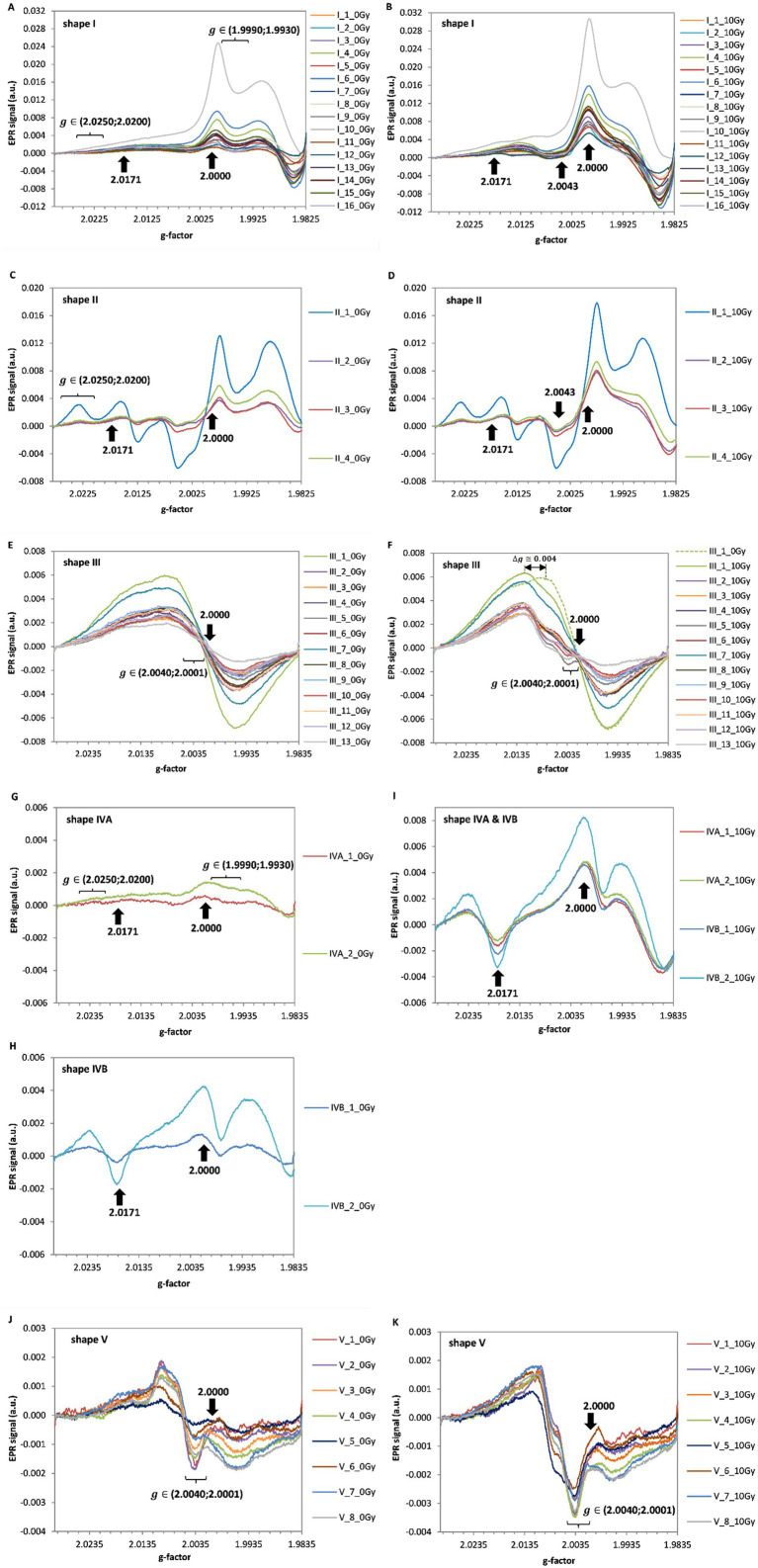
EPR spectra of the five line shapes classified in the 45 screens. The spectra before irradiation (0 Gy, at left) and after irradiation by 10 Gy (at right) are shown for: shape I **(A,B)**, shape II **(C,D)**, shape III **(E,F)**, shape IV **(G–I)** and shape V **(J,K)**, respectively. The arrows and the marked ranges of g-values indicate positions crucial in designation of the spectra to one of the I-V types, according to the sorting algorithm from Figure 1. The shift in g-value of the maximum in spectrum of the irradiated sample relatively to the BG spectrum is marked by the horizontal arrow **(F)**.

EPR spectra of all 45 touch screens tested, unirradiated and irradiated with a dose of 10 Gy, are presented in Figures 2A–K. In all types of the tested glasses the irradiation induced changes in their EPR signals.

The line-shape III (Figures 2E,F) is similar to previously reported spectra for watch glass (25, 26, 31), and window glass (23), which are known to be made of soda lime glass (22, 23, 26, 32), the mineral glass of type I obtained from mobile phones and watches is similar to that reported by Bassinet et al. (10) and to the type I spectra of LCD and touch screen presented by Trompier et al. (28). Among the five types of line-shapes measured in mobile phones by Trompier et al. (28), only this one similarity was found, which is probably related to the continuous changes in manufacturing of the screen glasses over passing years - in this article we present results from touch screens of devices produced later, between 2015–2019.

Figure 2F shows exemplary EPR spectra of irradiated and unirradiated samples of line-shape III (sample III_1). Similarly to Bassinet et al. (10), a broad and intense EPR signal of type I (according to their classification) was observed at g = 2.002. A shift in the maximum of the spectrum measured after irradiation with respect to the spectrum of unirradiated samples was observed. Both signals overlap at the minimum position. This is consistent with similar observations of Kortmis et al. (32, 33) for soda lime glass, who used magnitude of this shift in the spectrum to improve accuracy of dosimetry in a low-dose range.

**Figure 3:**
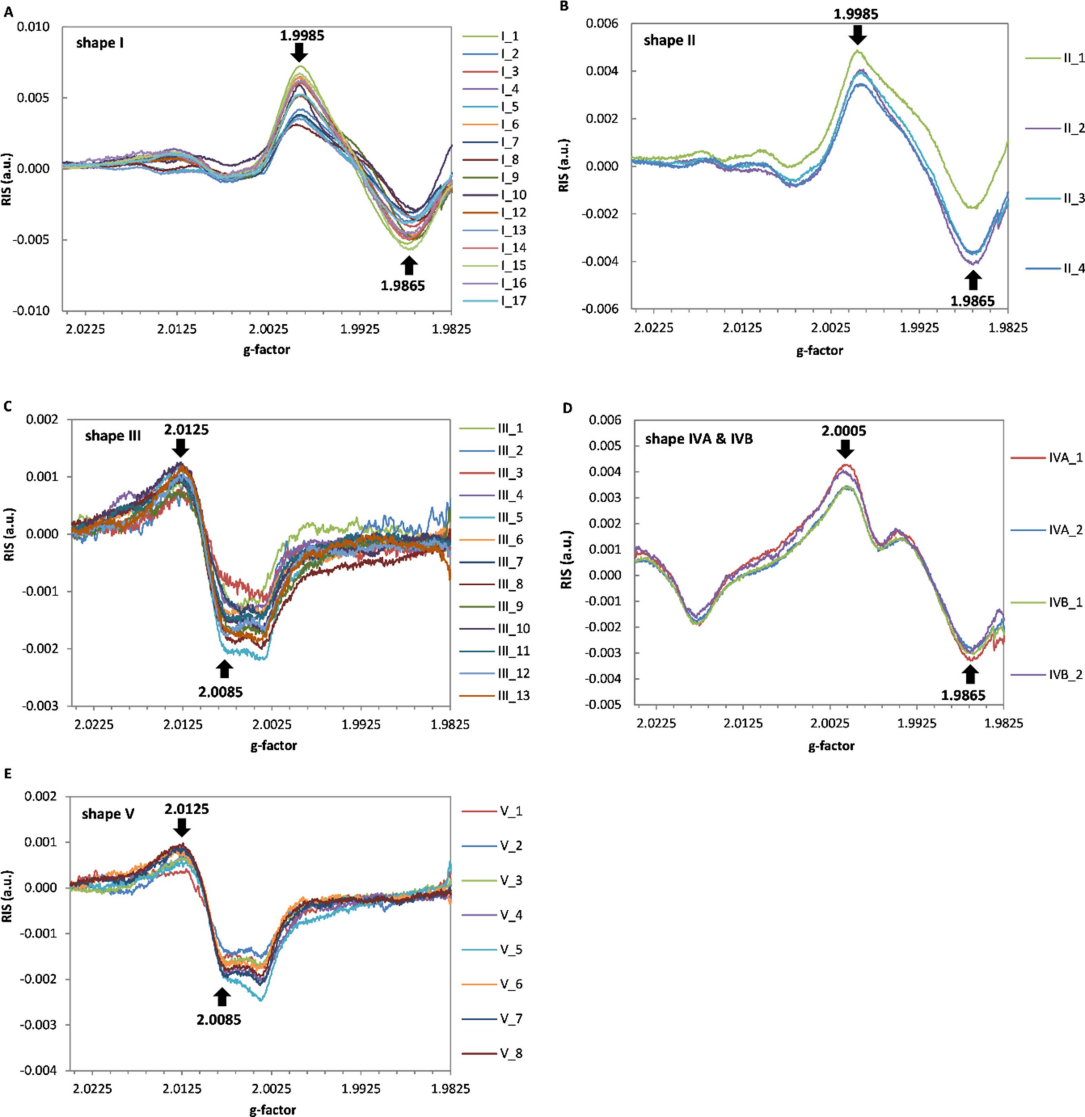
The radiation induced signals (RIS) in types I-V **(A-E)**, respectively. The radiation sensitivity was determined as peak-to-peak amplitude between the spectral positions marked by the arrows.

Figure 3 presents the dependence of the extracted radiation induced signals (RIS) for all measured devices. Three different line-shapes of the RIS components can be distinguished in all samples for type I and II, III and V and IV. However, as can be noticed in Figures 2A,C,E,G,H,J the respective BG signals for all individual types of glass differed from each other. On the basis of Figures 3A–E, the radiation sensitivity was determined, defined as sensitivity of the amplitude of the EPR spectrum to the dose, i.e., the peak-to-peak amplitude of the RIS per 1 Gy. It was measured for shapes I and II between the values at g = 1.9865 and g = 1.9985, for shape III and V between the values at g = 2.0085 and g = 2.0125 and for shape IV - between the values at g = 1.9865 and g = 2.0005. Measured average values of the radiation sensitivity and their standard deviations (in arbitrary units) were: for shape I - 0.00096 ± 0.00020; for shape II - 0.00074 ± 0.00007; for shape III - 0.00026 ± 0.00004 for shape IVA & IVB - 0.00069 ± 0.00006 and shape V - 0.00027 ± 0.00004.

It should be emphasized, that line shapes of the RIS signals are clearly radiation-specific, i.e., are different from the BG signals of the respective non-irradiated samples for all types of spectral line shapes (I–V) discussed, which enables their use for dose assessments, in particular using the numerical decomposition/deconvolution method (24, 25).

In order to check whether the elemental composition of the tested glasses is one of the parameters differentiating the types of shapes recognized by the sorting algorithm, the following analysis was performed. Eight dimensions were taken into account in the clustering analysis - the radiation sensitivity (RS) and the content of seven chemical elements: silicon (Si), potassium (K), magnesium (Mg), aluminum (Al), sodium (Na), calcium (Ca), phosphorus (P). This way each sample can be presented as a point in 8-dimensional space.

**Figure 4:**
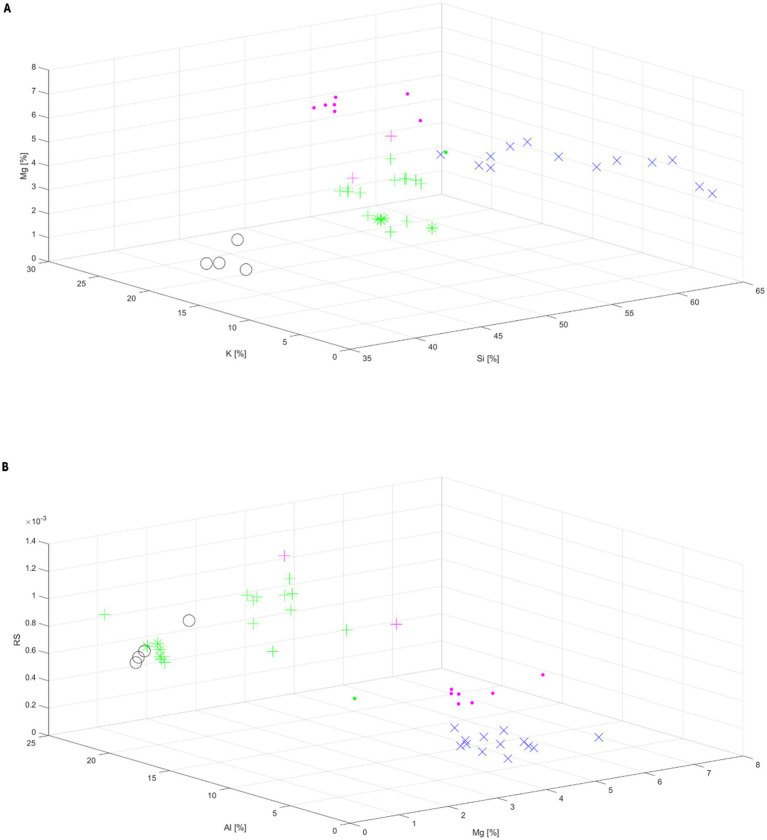
Clustering of distribution of the sample points represented by three features: content of Mg, K and Si **(A)** and radiation sensitivity (RS), content of Al and Mg **(B)**. The five types of line-shapes were marked as: shape 1 – (+), shape 2 – (*), shape 3 – (×), shape 4 – (○), shape 5 – (•).

The clustering of the points with c-means algorithm recognized four groups in the data points (29, 30). This is an unsupervised clustering algorithm, in which the groups are found by assuming their centres and calculating the distances of all points to the centres. Finally after clustering, a three-dimensional space with the labeled points was created. Two of the clustering results, with starting number of groups taken as 5, are presented on Figures 4A,B.

The groups in Figures 4A,B are labeled by different colors – black, blue, green and pink. In both cases, the algorithm distinguished 4 groups: the 1st marked as blue crosses (×) – all points representing shape III; the 2^nd^ marked as black circles (○) – all points for shape IV; the 3^rd^ marked as pink dots (•) – 7 belonging to the glasses of shape V and two pluses (+) belonging to shape I; the 4^th^ marked in green – 14 pluses (+) belonging to shape I, all points of shape II marked by stars (*) and 1 dot marking a sample of shape V. With one exception, the algorithm separated glasses of type V and combined most samples of type I and all samples of type II into one group.

Figures 4A,B show that the lowest Si content (at the level of about 40%) was detected in glasses exhibiting the type IV of line-shape. Samples representing line-shape III contain the largest amount of Si – 57.7-64.2% and the smallest amount of aluminium 1.9–5.6% (Figure 4A) and show the lowest radiation sensitivity at an average level of about 0.00026. Furthermore, the data presented in Figure 4B show that the lowest magnesium content is observed for samples having high radiation sensitivity (shape I, II and IV), and samples having low radiation sensitivity (shape III and V) contain magnesium at high level.

### 3.1 The effects of temperature and UV on EPR signals in screen glass

**Figure 5:**
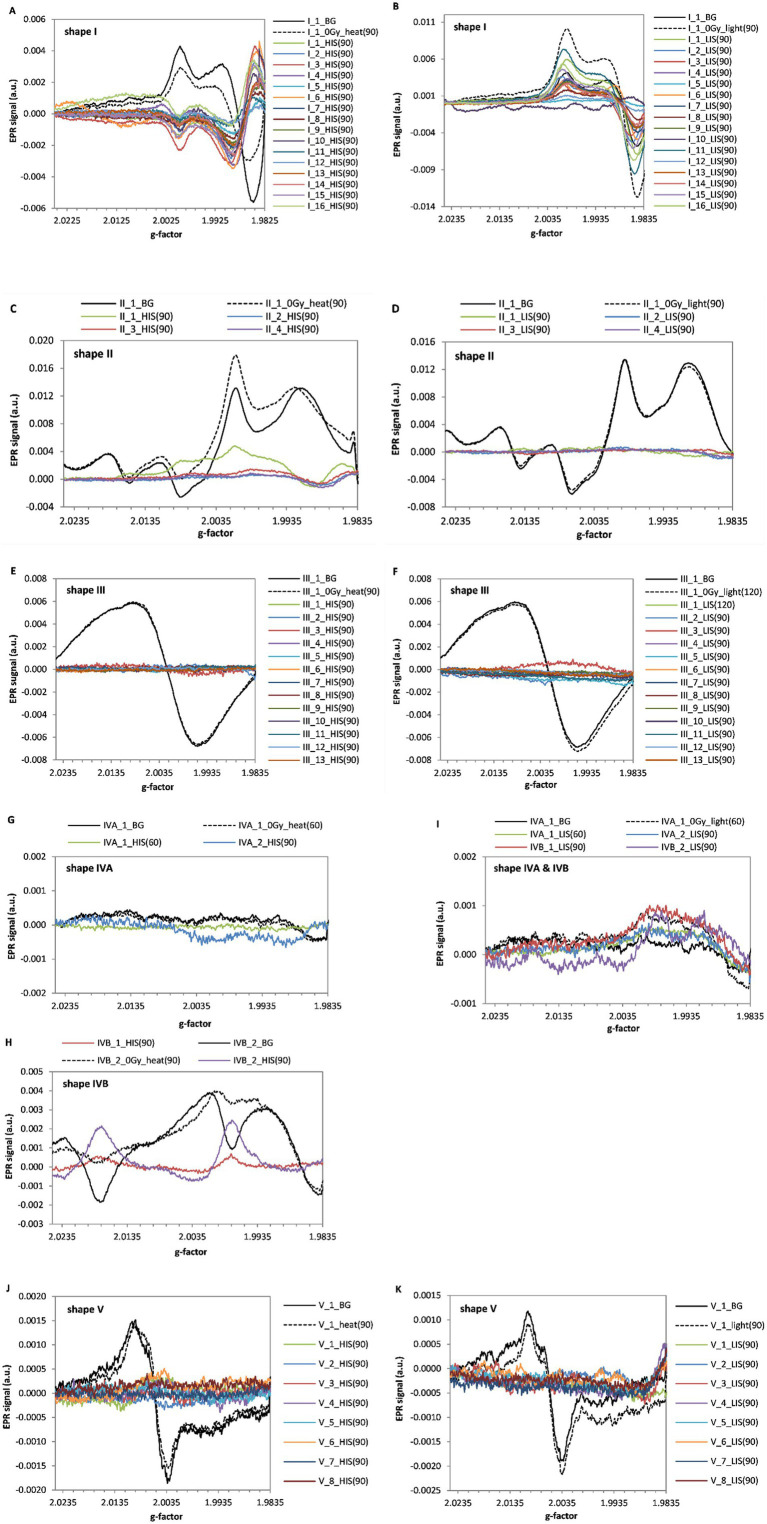
EPR spectra of extracted HIS and LIS components of the five types of line-shapes. The spectra before irradiation (BG), after heating at 200 °C (for periods in minutes specified in parentheses) and the extracted HIS components are shown for: shape I **(A)**, shape II **(C)**, shape III **(E)**, shape IV **(G,H)** and shape V **(J)**, respectively. The spectra before irradiation (BG), after illumination by UV (for periods in minutes specified in parentheses) and the extracted LIS components are shown for: shape I **(B)**, shape II **(D)**, shape III **(F)**, shape IV **(I)** and shape V **(K)**, respectively.

Figure 5 presents spectra contributing to the overall EPR signal: native (BG), heat-induced (HIS) and light-induced (LIS) in all tested samples. The spectral components were determined experimentally (separately for each samples of five types the glass): the BG was measured in samples neither exposed to ionizing radiation nor illuminated by light other than in normal laboratory conditions, the LIS spectra were obtained by subtracting spectra of the UV illuminated, unirradiated samples and their BG spectra, and the HIS spectra were obtained by subtraction of the BG spectra from those measured in samples annealed at 200 °C. All those spectra were determined separately and potentially can be used as model spectra in numerical decomposition to extract the RIS components for dosimetry.

The occurrence of the effects of light from the CLEO lamp (LIS) and annealing at 200 °C (HIS) on the native signal of selected samples representing each of the five spectral line-shapes is presented in Table 3.

**Table 3:** Properties of the five types of group of screen glasses regarding induction of spectral changes by UV (induction of LIS) and by heating at 200 °C (induction of HIS).

Spectral component	shape I	shape II	shape III	shape IVA	shape IVB	shape V
HIS	Yes	Yes	No	No	Yes	No
LIS	Yes	No	No	Yes	Yes	No

**Figure 6:**
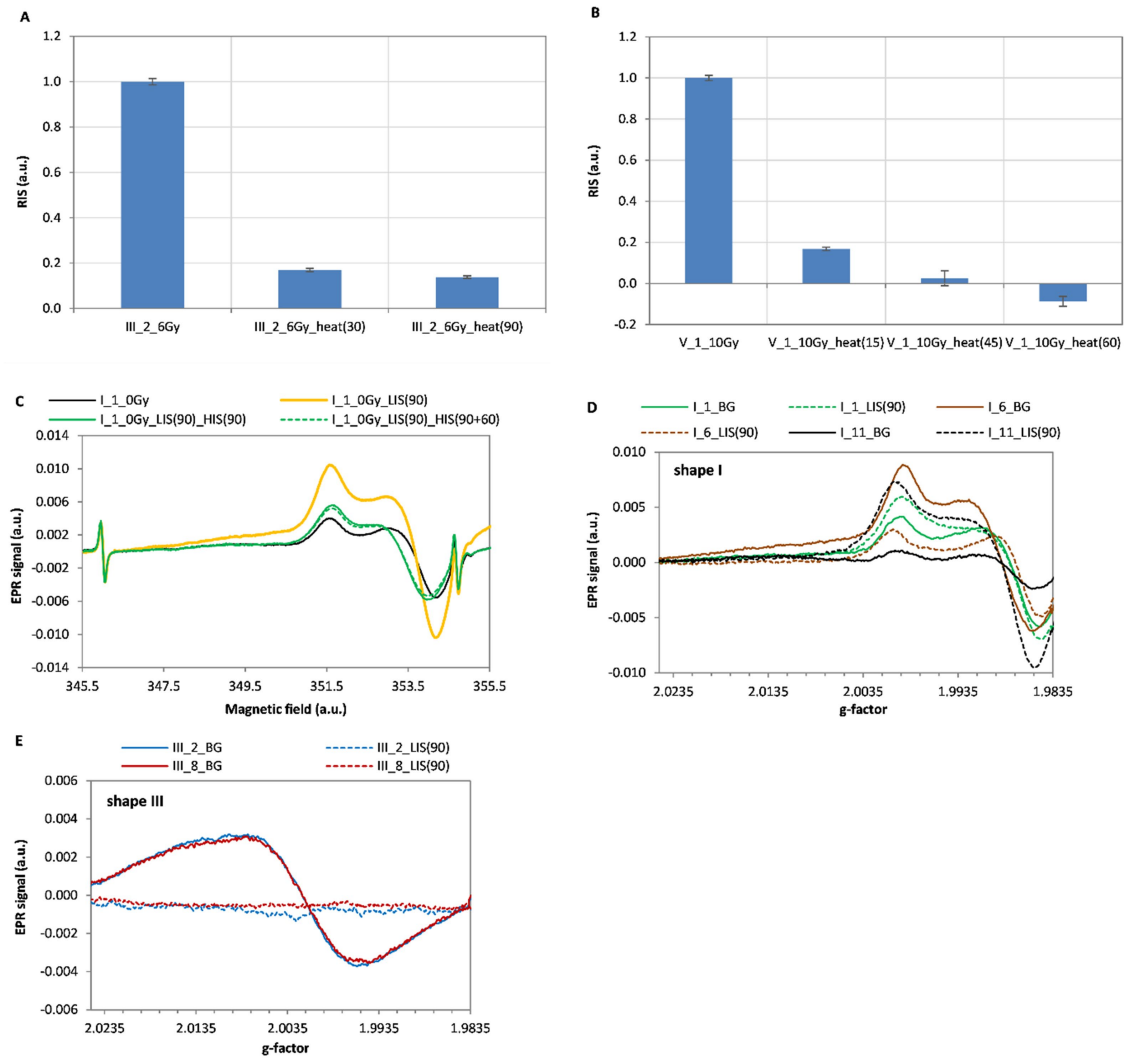
The decay of the RIS component vs. heating time for sample III_2 **(A)** and sample V_1 **(B)**. EPR spectra of unirradiated I_1 sample: BG – black line, sample exposed to 90 min UV light – yellow line, sample exposed to UV and annealed for 90 and 150 min – green solid and dotted lines, respectively **(C)**. Comparison of EPR spectra of unirradiated glass samples: native (BG) and the extracted light-induced (LIS) signals from different phones of the same model of **(D)** Samsung Galaxy Ace 4 (SM-G357FZ), **(E)** Microsoft Mobile RM-1127 (Microsoft Lumia 550). Samples marked as I_11 were obtained from new smartphones purchased for this project.

Figures 6A,B show the dependence of magnitude of the RIS component, identified in the samples’ spectra by numerical decomposition, on duration of the annealing at 200 °C for 30 and 90 min for sample III_2 (Figure 6A) and for 15, 45 and 60 min for sample V_1 (Figure 6B). The data presented in Figure 6A show that the 90 min of annealing at 200 °C reduces the RIS signal approximately tenfold. A similar result was obtained for sample V_1 after annealing for 45 min (Figure 6B). Those results and the lack of HIS in those glass types (Figures 5E,J) confirm applicability of the heating method (25) to recover the BG signal in an irradiated sample, which is necessary for a reliable dose reconstruction. The effect of heating at 200 °C on EPR signal of the sample I_1 not irradiated with ionizing radiation but exposed to UV light is shown in Figure 6C. Figures 6D,E show a comparison of BG and LIS signals of glasses from the same phone models representing the same categories of spectral line shapes. Figure 6D compares three Samsung Galaxy Ace 4 phones (marked as I_1, I_6 and I_11), which were classified as type I. Phone I_11 was brand new, purchased for the needs of this project, while the other two were used by their owners. The lowest BG signal intensity was observed for the sample I_11, i.e., from the new, non-used phone. The light-induced signal (LIS) in this phone was the largest. The other two samples show much higher BG intensity and smaller LIS induced by the same UV exposure. These differences suggest a potential previous exposure of those used screens to UV component in sunlight during daily usage of these phones, which is consistent with the results of research conducted by Juniewicz et al. (21) showing a saturation effect of the LIS with even short (dozens of minutes) exposures of the glass to UV.

For shape III, the BG signal was compared for two used Microsoft Mobile RM-1127 phones marked as III_2 and III_8 (Figure 6E). In this case, no significant differences in the intensity or shape of the spectral line were observed between the tested samples, which confirms a lack of sensitivity of these types of glass to UV, which is concurrent with our experimental data shown in Table 3.

## 4 Discussion

The presented categorization algorithms proved to be successful in differentiation all examined spectra into five groups having different properties with respect to effects of UV and/or heating on the samples’ EPR spectra. Those two effects are important in assessment of the applicability of glass for retrospective dosimetry. Namely, the occurrence of LIS imposes the necessity of including the model LIS signal in spectral decomposition in extracting the dosimetric RIS component. Only then one can continue further dosimetric analysis, however a possibility of light-induced bleaching of the RIS component still must be taken into account. The occurrence of HIS is a serious contraindication, if one wants to reconstruct the original BG signal from already irradiated sample by annealing. However, if heating only increases or reduces intensity of the native BG signal (i.e., the HIS has the same line-shape as the native BG) the procedure of numerical decomposition should account for this effect. Consequently, reconstruction of the correct value of the RIS component should be possible using the spectrum from heated sample for model BG in spectra decomposition instead of the real native BG.

The presented sorting algorithm proved to be effective in grouping of our spectra, however its accuracy is sensitive to spectra acquisition parameters affecting the line-shape, in particular modulation amplitude, spectra filtering (if applied) and baseline correction. Therefore, some adjustments in this algorithm can be necessary for proper categorization of spectra measured with different spectrometer or with different acquisition parameters. This categorization can be automatized in Excel or any other data analysis program.

All tested glasses showed a specific RIS signal (Figure 3), i.e., different in shape from the native background (BG). This feature enables dose reconstruction based on spectra decomposition. The RIS component is similar for types I and II and also for types III and V. However, the sensitivity to radiation is lower for type II than I, and is similar in glasses assigned to types III and V.

Shape-lines type III and IV are exhibited by glasses having a unique elemental composition characterized by the presence of calcium and phosphorus, respectively (Table 1). The line-shape I was dominant among the glasses we tested (about 36%). Significant differences in intensity of the BG spectra assigned to shape I, observed in samples from three various phones of the same model with the smallest BG measured in brand new phone (Figure 6D), suggest difference in previous exposure of those phones to sunlight. This hypothesis is confirmed by the presence of a specific light-induced spectral component (LIS) observed in sample I_1 (Figure 5B; Table 3). A potential exposure of the glass to UV (e.g., to direct sunlight) is an important factor affecting reliability of dose reconstructions, however, it was observed that annealing at 200 °C of an unirradiated sample, but illuminated with UV light, causes a decrease in the EPR signal intensity by destroying the LIS component (Figure 6C).

Glasses exhibiting shape-line III are very common in the human environment - as window glasses, watch glasses, phone displays (29% of the glasses we examined), car windows glass, light bulbs (32). All of them show a weak but specific RIS signal and a shift of the spectra maximum toward higher g values after irradiation. Determination of this shift in relation to the unirradiated signal can improve accuracy of dosimetry in a low-dose range when using methods, which are not based on decomposition of the spectra to BG and RIS components (32).

Glasses representing type V of spectral line are the third largest group (18%) tested in this project. A common feature for both types III and V is similar shape of the radiation-induced signals (RIS) and a comparable sensitivity of their EPR signal to ionizing radiation. Moreover, BG signal of glasses from types III and V is resistant to high temperatures >200 °C. Therefore, annealing of those glasses allows for elimination of the RIS signal and, consequently, recovery of the native signal in samples irradiated during an accident as shown by previous research of Marciniak et al. (25). Furthermore, for both of these shapes no effect of UV light on the native background was observed, which is advantageous for retrospective, accidental dosimetry. Nevertheless, the effect of light on magnitude of the RIS has to be examined for reliability of dosimetry.

For line-shape of type IV a LIS component was observed (Figure 5I; Table 3). Since the influence of UV exposure is an important factor affecting accuracy of dose reconstructions, additional studies are necessary to characterize UV influence on RIS and on the measured doses. For non-irradiated samples, two subgroups of this spectral shape-line (IVA and IVB) were distinguished, but they show the same shape of their RIS signal.

Due to the small number of samples assigned to types II and IV, the obtained results should be confirmed on a larger population. This would allow for verification of the presented here preliminary results and formulation of recommendations for use of those glasses in dosimetry.

Glasses type I and IV are sensitive to UV (Table 3), which is a serious disadvantage for their potential dosimetric applications. Additional research is necessary in order to analyse a possibility of reliable determination of the dosimetric signal (RIS) under the presence of the confounding LIS component. Previously, it was shown by us (21), that the light for UV lamp causes similar effects in screen glasses to sunlight, therefore our conclusions in this manuscript regarding the UV light can be qualitatively extrapolated to potential effects of environmental UV (sunlight).

Results of the clustering analysis show interesting correlations between elemental composition of the glasses and their assignment to different spectral types. More specific conclusions from this observation at the present state of research could not be drawn; this study will be continued.

Summarizing, it can be stated that glasses showing line-shape III and V of the EPR spectrum aspire to be a better material for a personal dosimeter than the other types due to their insensitivity to UV light (lack of LIS) and stability of the BG signal at high temperatures (lack of HIS). The latter feature allows for application of the heating method (25) for reconstruction of the BG signal – a necessary step in EPR dosimetry.

One can expect, that sensitivity of detection and accuracy in quantitative determination of the dosimetric spectral component (RIS) may considerably improve, when instead of our method of numerical extraction of the RIS from the experimental spectra (the Excel’s Reglinp procedure), more sophisticated novel methods of spectra analysis are applied, e.g., using deconvolution based on machine learning (34). Nevertheless, regardless the applied methods of spectra analysis, for practical reasons an initial assessment of applicability of given glass for dosimetry, possible to perform quickly and reliably using our classification algorithm is helpful and important. The presented categorization of the spectra shapes, which relates line-shape features to sensitivity of glasses to UV, high temperature and their elemental composition, the development of automatic sorting algorithm created for categorization of EPR spectra can be the first step toward creating a database containing numerous EPR spectra from various phones, together with the above-mentioned features of glasses, useful in EPR dosimetry. Such database, constantly updated with data from new phones, would allow for a quick assessment of applicability of a given glass for retrospective dosimetry. It would be helpful in selecting the appropriate dose reconstruction method. It should also contain downloadable model BG spectra for various glasses, which are indispensable in determination of the dose in glasses, for which the annealing method cannot be applied. In consequence, it may significantly facilitate and shorten the dosimetry procedure, which is extremely important in a scenario of realistic radiation accident.

## Data Availability

The raw data supporting the conclusions of this article will be made available by the authors, without undue reservation.
